# Transitory Fetal Skin Edema in a Pregnant Patient with a Mild SARS-CoV-2 Infection

**DOI:** 10.1155/2021/5552877

**Published:** 2021-03-16

**Authors:** Alicia Martínez-Varea, Julia Desco-Blay, Sagrario Monfort, María Hueso-Villanueva, Alfredo Perales-Marín, Vicente José Diago-Almela

**Affiliations:** Department of Obstetrics and Gynecology, University and Polytechnic La Fe Hospital, Avenida Fernando Abril Martorell 106, 46026 Valencia, Spain

## Abstract

**Background:**

Vertical transmission of the Coronavirus Disease 2019 (COVID-19) is still controversial. Additionally, the consequences of the infection during pregnancy in the offspring also are unknown.

**Case:**

A transitory fetal skin edema and polyhydramnios have been demonstrated by ultrasound in a pregnant patient with COVID-19 after a negative RT-PCR for SARS-CoV-2. The fetal findings presented a spontaneous resolution in utero, and abnormal findings were not found in the newborn.

**Conclusion:**

Women who have undergone SARS-CoV-2 infection during pregnancy should receive a subsequent appropriate follow-up in order to clarify the fetal consequences of the novel coronavirus, if any.

## 1. Introduction

The ongoing pandemic is caused by the severe acute respiratory syndrome coronavirus 2 (SARS-CoV-2), which was initially identified in Wuhan, China, at the end of 2019 [1]. Due to its remarkably contagious behavior [2], the novel coronavirus has infected over 114 million people worldwide [3]. Mainly transmitted through respiratory droplets, it leads to pneumonia [4], and the clinical course of severe or critical SARS-CoV-2 infection in hospitalized pregnant women may be shorter than in nonpregnant patients [5].

Not only is vertical transmission of the Coronavirus Disease 2019 (COVID-19) still controversial [6–18], but the fetal consequences of the infection during pregnancy also are unknown [7, 19, 20]. It has been recently reported fetal transient skin edema in two pregnant women while they showed positive reverse-transcription polymerase chain reaction (RT-PCR) test results for SARS-CoV-2 infection [21]. At our tertiary hospital, a transitory fetal skin edema and polyhydramnios were demonstrated by ultrasound in a pregnant patient with a mild infection after a negative RT-PCR for SARS-CoV-2.

## 2. Case Report

A 22-year-old gravida 2, para 1 woman at 28 3/7 weeks' gestation presented to the emergency department at La Fe University and Polythecnic Hospital (Valencia, Spain) with a 3-day history of mucus and shortness of breath. Neither fever nor cough were shown. Her partner had been admitted into the hospital 24 hours before due to pneumonia and a positive RT-PCR test for SARS-CoV-2. The pregnancy was spontaneously conceived and uneventful, with a low risk first trimester screening for trisomies 21, 18, and 13, as well as a normal fetal anatomy visualized at the 20 weeks' morphological ultrasound. Physical examination revealed a body mass index, temperature, blood pressure, respiratory rate, and hemoglobin saturation by pulse oximetry within normal limits. Blood tests showed a D-dimer level of 1680 ng/ml, and the remainder parameters were within the normal range. The patient tested positive for SARS-CoV-2 infection on RT-PCR of nasopharyngeal swabs. A chest radiograph revealed the absence of abnormal images, and the fetal ultrasound was reassuring. Hence, she was discharged and her well-being was assessed every 48-72 hours by phone calls. The symptoms of infection lasted for a total of 10 days, and the RT-PCR for SARS-CoV-2 became negative at 32 6/7 weeks. IgG and IgM antibodies were negative in maternal serum at 32 6/7 weeks.

**Figure 1:**
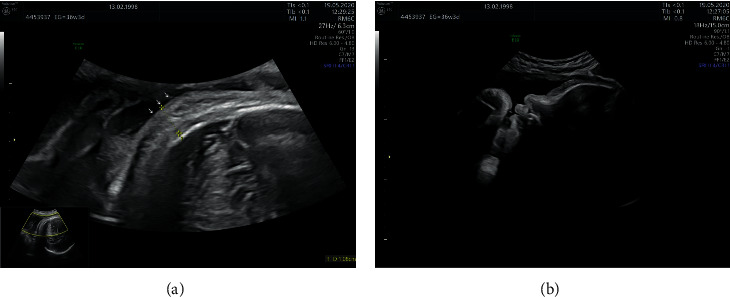
(a) A transitory fetal skin edema (11 mm) was visualized in the axial plane of the fetal head by ultrasound at 36 3/7 weeks of pregnancy. (b) Sagittal view of the transitory fetal head skin edema at 36 3/7 weeks of pregnancy.

**Figure 2:**
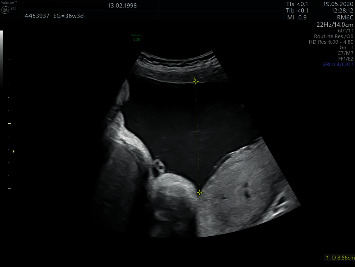
Polyhydramnios (maximum vertical pocket 86 mm at 36 3/7 weeks of pregnancy).

The fetal ultrasound at 35 0/7 weeks gestation revealed an estimated fetal weight in accordance with the gestational age (2645 g) and a maximum vertical pocket of amniotic fluid of 81 mm (Table 1). The subsequent follow-up with serial ultrasounds revealed a transitory fetal skin edema (11 mm in the axial plane of the head at 36 3/7 weeks, Figures 1(a) and 1(b); and 4 mm at 37 3/7 weeks gestation), as well as polyhydramnios (maximum vertical pocket 86 mm at 36 3/7 weeks, Figure 2; and 88 mm at 37 3/7 weeks gestation). Peak systolic velocity remained within normal limits. Maternal serology was negative, and the glucose level was normal. Both developed a spontaneous resolution. IgG and IgM antibodies remained negative in maternal serum at 38 3/7 weeks. In the ultrasound at 38 3/7 weeks gestation, no fetal skin edema of the head was seen, the estimated fetal weight was 3500 g, and the maximum vertical pocket of amniotic fluid was 76 mm. The patient underwent a spontaneous vaginal delivery at 39 4/7 weeks of pregnancy. The female newborn weighted 3700 g, associated an Apgar Score 10/10, an arterial pH 7.28, and a venous pH 7.33. Neither cord blood PCR nor antibodies were assessed. Both, the mother and the newborn, were healthily discharged 48 hours after birth.

## 3. Discussion

Pregnant women are particularly vulnerable to infections due to their relative immunosuppressed state and restricted cardiorespiratory capacity [22]. Nonetheless, it has not been described that pregnant women have a predisposition to SARS-CoV-2 infection or that those with the infection are more susceptible to develop severe pneumonia [23]. The infection is severe or critical in 9% and 5% of pregnant women, respectively [24], and these percentages are similar to those of nonpregnant patients [25].

Regarding the adverse maternal and perinatal outcomes associated with COVID-19, preterm delivery is the most common adverse pregnancy outcome among women with severe disease [5, 23, 26]. As far as the risk of vertical transmission is concerned, although it remains controversial [6–18], placental infection by SARS-CoV-2 has been recently demonstrated [6, 16, 18]. Two cases of fetal transient skin edema during the second trimester of pregnancy in women with COVID-19 have been recently described, being the pregnancy outcome unknown given that the patients were still pregnant at the time of the publication [21]. The authors hypothesized that these findings might constitute the expression of fetal infection or the consequence of the maternal infection in the fetal physiology [21]. The current case report describes a transitory fetal skin edema associated with polyhydramnios in a pregnant woman with COVID-19 after the RT-PCR testing for SARS-CoV-2 was negative. The fetal findings presented a spontaneous resolution in utero, and abnormal findings were not found in the newborn. Neither RT-PCR testing nor IgG and IgM antibodies were performed to the neonate. Although causes of fetal hydrops such as gestational diabetes or other viral infections were ruled out in our patient, further fetal assessment by ultrasound in pregnant women with COVID-19 are required to elucidate whether these fetal findings are common and if they entail any consequence in the offspring.

In conclusion, these findings support the need of a close monitoring of women who have undergone SARS-CoV-2 infection during pregnancy in order to completely understand the fetal consequences of the novel coronavirus. Additionally, it remains essential the assessment of the umbilical cord blood of the newborn, either PCR or antibodies, from those mothers who have undergone COVID-19 during pregnancy.

## Figures and Tables

**Table 1:** In-person follow-up of the pregnant woman after the PCR for SARS-CoV-2 was negative.

Gestational age at ultrasound (weeks)	Estimated fetal weight (g)	Maximum vertical pocket of amniotic fluid (mm)	Peak systolic velocity (cm/seg)	Fetal anomalies
35 0/7	2645	81	57	None
36 3/7	2789	86 (Figure 2)	54	Fetal skin edema (11 mm) was visualized only in the head (Figure 1)
37 3/7	3371	88	55	Fetal skin edema (4 mm) in the axial plane of the head
38 3/7	3500	76	56	None
